# Symbiont Community Changes Confer Fitness Benefits for Larvae in a Vertically Transmitting Coral

**DOI:** 10.1002/ece3.70839

**Published:** 2025-01-12

**Authors:** Daniel Olivares‐Cordero, Courtney Timmons, Carly D. Kenkel, Kate M. Quigley

**Affiliations:** ^1^ Department of Biological Sciences University of Southern California Los Angeles California USA; ^2^ Minderoo Foundation Perth Western Australia Australia; ^3^ James Cook University Townsville Queensland Australia; ^4^ University of Western Australia Crawley Western Australia Australia

**Keywords:** coral reefs, heat stress, Symbiodiniaceae, symbiosis, transgenerational acclimatization

## Abstract

Coral reefs worldwide are threatened by increasing ocean temperatures because of the sensitivity of the coral‐algal symbiosis to thermal stress. Reef‐building corals form symbiotic relationships with dinoflagellates (family Symbiodiniaceae), including those species which acquire their initial symbiont complement predominately from their parents. Changes in the composition of symbiont communities, through the mechanisms of symbiont shuffling or switching, can modulate the host's thermal limits. However, the role of shuffling in coral acclimatization to heat is understudied in coral offspring and to date has largely focused on the adults. To quantify potential fitness benefits and consequences of changes in symbiont communities under a simulated heatwave in coral early life‐history stages, we exposed larvae and juveniles of the widespread, vertically transmitting coral, *Montipora digitata,* to heat stress (32°C) and tracked changes in their growth, survival, photosynthetic efficiency, and symbiont community composition over time relative to controls. We found negative impacts from warming in all fitness‐related traits, which varied significantly among larval families and across life‐history stages. Larvae that survived heat exposure exhibited changes in symbiont communities that favored symbionts that are canonically more stress tolerant. Compared to larvae, juveniles showed more rapid mortality under heat stress and their symbiont communities were largely fixed regardless of temperature treatment, suggesting an inability to alter their symbiont community as an acclimatory response to heat stress. Taken together, these findings suggest that capacity for symbiont shuffling may be modified through ontogeny, and that the juvenile life stage may be less flexible and more at risk from climate warming in this species.

## Graphical abstract

We quantified the fitness consequences of changes in symbiont communities under a simulated heatwave in early life‐history stages of *Montipora digitata* by tracking changes in their growth, survival, photosynthetic efficiency, and symbiont community composition relative to controls. Surviving larvae exposed to heat exhibited changes in symbiont communities that favored types that are canonically more stress tolerant. We found negative impacts with warming in all fitness‐related traits, which varied significantly among larval families and life stages. Surviving larvae exposed to heat exhibited changes in symbiont communities that favored types that are canonically more stress tolerant. Compared to larvae, juveniles showed more rapid mortality under heat stress and their symbiont communities were largely fixed regardless of temperature treatment, suggesting an inability to alter their symbiont community as an acclimatory response to heat stress.

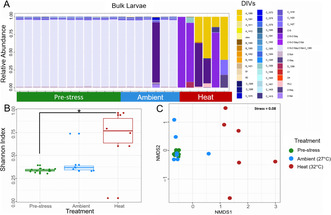

## 1 Introduction

Across the tree of life, symbiosis fuels biodiversity and many species engage in these life‐long partnerships to increase their fitness through mechanisms like nutrient exchange, shelter, or chemical defenses (Sachs et al. 2004). However, symbiotic relationships are not always equitable or stable over ecological timescales as observed in the nutritional exchange underpinning life on coral reefs, the coral‐algal symbiosis, which is particularly sensitive to environmental perturbation (Kiers et al. 2003; Kiers et al. 2010; Davy, Denis, and Weis Virginia 2012). Ocean warming currently represents the greatest threat to the persistence of coral reefs globally, and as the main driver of warming, human‐induced climate change is increasingly leading to more severe and frequent bleaching and mortality events on reefs (van Woesik et al. 2022). Coral bleaching is defined as the breakdown of the partnership between photosynthetic dinoflagellate symbionts (Symbiodiniaceae) and their coral hosts from environmental stress. Generally, this can be caused by high and persistent temperature and high light (van Woesik 2001). If stressful conditions persist, this can result in mortality of the host due to the loss of symbiont‐derived nutrition or destruction of host tissues (Glynn 1993). One key driver of determining bleaching thresholds in coral individuals and populations is the composition of Symbiodiniaceae hosted by the coral (Baker 2004; Berkelmans and Van Oppen 2006).

Dinoflagellates in the family Symbiodiniaceae are classified into 15 genera with most capable of forming a symbiosis with coral (LaJeunesse et al. 2018; Yorifuji et al. 2021; Nitschke et al. 2022). Corals vary in the degree of specificity in their symbiotic partnerships (Sampayo et al. 2016; Elder et al. 2023) and the range of potential Symbiodiniaceae partners is diverse (LaJeunesse et al. 2018). Some hosts maintain simultaneous relationships with multiple symbionts, whereas others are more specific (Baker 2003; Little, van Oppen, and Willis 2004; Baird et al. 2007; Howells et al. 2020; Davies et al. 2023). Importantly, differences in these communities drive variation in host fitness, including heat and light tolerance, and growth rates (Putnam et al. 2012; Swain et al. 2017; Cunning, Silverstein, and Baker 2018; Matsuda et al. 2022; Davies et al. 2023). For example, an increase in the relative abundance of *Durusdinum trenchii* (formerly D1a, LaJeunesse et al. 2018) in three coral species resulted in increased heat tolerance (Cunning, Silverstein, and Baker 2018).

In addition to their phylogenetic diversity, the coral‐Symbiodiniaceae symbiosis can be modified through the ecological mechanisms by which corals acquire and dynamically regulate their symbiont communities which also influences thermal resistance and resilience of the holobiont. There are two main mechanisms by which symbiont communities change *in hospite*, namely shuffling and switching. Symbiont shuffling refers to changes in the relative abundance of community members already in residence (Baker 2001; Baker et al. 2004). Generally, this involves a reduction in the abundance of a dominant symbiont due to an environmental change which provides an opportunity for a numerically rarer symbiont(s) to increase in relative abundance (Quigley et al. 2022). This process should, by definition, result in an increase in host fitness and may be adaptive (Baker et al. 2004). Switching refers to the ability of a host to replace an existing symbiosis by selecting for a novel partner from the environment (Sørensen et al. 2021). Increased abundance of symbionts in the opportunistic and generally stress‐tolerant genus *Durusdinium* is the canonical example of shuffling following heat stress (Berkelmans and Van Oppen 2006; Quigley et al. 2022), again emphasizing the role of symbionts in reef resilience (Berkelmans and Van Oppen 2006; Quigley et al. 2022). However, our knowledge of the functional relevance of shuffling and switching is generally limited to adult coral and has only been examined in early life‐history stages in a few studies (Quigley, Willis, and Kenkel 2019; Terrell et al. 2023).

Symbiont communities in adult corals are also influenced by the mode of symbiont acquisition (Fabina et al. 2012). In corals, there are three known mechanisms for symbiosis initiation: vertical, horizontal, or mixed‐mode, with the majority of coral employing horizontal transmission (Quigley et al. 2018; Baird, Guest, and Willis 2009). Horizontally transmitting corals must acquire their algal symbionts from the environment each generation. Vertical transmitters, on the other hand, obtain their symbiont community from a maternal source, often through the infection of oocytes before fertilization or planula during gestation (Davy and Turner 2003; Hirose and Hidaka 2006; Padilla‐Gamiño et al. 2012). Mixed mode transmission refers to the ability for corals to inherit their symbiont community from a maternal source with the additional ability to acquire symbionts from the environment during development (Ebert 2013). Thus far, coral species have generally been categorically described as either vertical or horizontal transmitters (Baird, Guest, and Willis 2009), although mixed mode transmission was recently described in a canonical vertical transmitter (Quigley et al. 2018).

A better understanding of transmission mode is critical because it affects the long‐term fidelity of the symbiotic association (Ebert 2013; Quigley et al. 2018; Dixon and Kenkel 2019). Vertically transmitted symbioses are generally thought to be co‐evolved associations, in which the diversity of symbionts in the host coral is lower (Fabina et al. 2012), and the ability of the symbiont to live outside the host is restricted (Krueger and Gates 2012). However, in the vertically transmitting coral *Montipora digitata*, symbiont communities in offspring are more diverse compared to adults (Quigley, Willis, and Bay 2017), and alterations in symbiont community composition in adults due to stress are reflected in oocytes, supporting the potential for transgenerational inheritance of shuffled algal communities over time (Quigley, Willis, and Kenkel 2019). This suggests that vertically transmitted symbiont communities are more flexible than originally thought, and dynamic shifts in the complement of algal symbionts passed on to offspring may confer fitness benefits in variable environments (Björk et al. 2019).

Taken together, there is now evidence that flexible symbiotic partnerships may confer greater adaptive and acclimatory potential on the coral holobiont (Torda et al. 2017). However, the majority of our current understanding regarding the fitness impacts of flexible symbiont associations comes from studies on adult life stages (Baker 2001; Berkelmans and Van Oppen 2006; Mieog et al. 2007). Larval life stages of marine invertebrates have higher energetic demands (Pechenik 1999) which are further exacerbated by heat stress (Przeslawski, Byrne, and Mellin 2015). For example, coral larvae show increased respiration rates, decreased survival rates and decreased photosynthetic efficiency (Fv/fm) under heat stress (Putnam et al. 2013; Ross et al. 2013). Furthermore, heat‐induced differences in energetic demands of coral larvae can also vary across different family and population‐based crosses (Dixon et al. 2015; Zhang et al. 2019, 2023), underscoring that larval energetic costs to heat stress have a heritable basis. At the molecular level, coral larvae respond to heat stress with decreased expression profiles of heat‐stress responsive genes, changes in oxidoreductase activity, and cell death (Rodriguez‐Lanetty, Harii, and Hoegh‐Guldberg 2009; Polato et al. 2010; Dixon et al. 2015). At the level of symbiosis, a switch from immune suppression to immune activation was observed upon initiation of symbiosis in heat‐stressed larvae of a horizontally transmitting, *Acropora digitifera* which coincided with reduced larval survival when initiating symbiosis with their dominant symbiont (Kitchen et al. 2022). However, *Acropora tenuis* larvae subjected to heat stress were observed to have greater survivorship rates when exposed to mixed communities of symbionts in equal portions (*Cladocopium* sp., *Durusdinium* sp., *Fugacium* sp., and *Gerakladium* sp.) over a two‐week period (Matsuda et al. 2022). This makes our understanding of this nutritional endosymbiosis even more essential, particularly for vertically transmitting coral species as cooperation between host and symbiont is predicted to increase between both partners in this context (Douglas 1998; Sachs and Wilcox 2006; Nalepa 2020). In addition, the physiological consequences of the trans‐generationally inherited community shifts observed in oocytes on later coral life stages remain unknown (Quigley, Willis, and Kenkel 2019).

We exposed multiple cohorts of coral larvae and juveniles to heat stress and monitored changes in their physiology, survival and Symbiodiniaceae communities over time to evaluate the relationship between physiological metrics of fitness and symbiont community composition over coral ontogeny. In addition to reductions in photosynthetic efficiency of symbionts, and size and survival of the host, we show that heat stress in larval samples increased Symbiodiniaceae community alpha diversity through increasing abundances of *Symbiodinium*, *Durusdinium*, and *Fugacium* spp. Alternatively, heat‐stressed juveniles showed a limited capacity to change their symbiont communities. Finally, we show that increased community diversity of Symbiodiniaceae in maternal corals is reflected in the family of offspring and may have fitness consequences.

## 2 Materials and Methods

### 2.1 Sample Collection

*Montipora digitata* colonies were collected from Hazard Bay (S18°38.069′, E146°29.781′) and Pioneer Bay (S18°36.625′, E146°29.430′) at Orpheus Island 2 days before the full moon on the 29th of March 2018 and brought to the National Sea Simulator (Seasim) at the Australian Institute of Marine Sciences (AIMS). Colonies were collected under Permit Number: G12/35236.1. To minimize the collection of clonal *M. digitata* fragments, colonies were collected a minimum of 5–10 m apart laterally along the shore, given that clones tend to propagate shoreward with wave action (Heyward, pers. comm). Upon arrival to the Seasim, colonies were placed in outdoor tanks that were maintained at 27.5°C and filled with 0.2 μm filtered sea water (hereafter μm FSW) using National Sea Simulator technology which follows multiple bag and UV filtration. The colonies were then sorted into either the “fat‐finger” or “yellow‐spathulate” morphologies (Stobart 2000; Quigley, Willis, and Bay 2016).

### 2.2 Spawning and Fertilization Design

Colonies were monitored for spawning starting two nights before the full moon (29th of March) starting around dusk. Bundles were collected on the 6th of April, six nights past the full moon (31st of March 2018). Egg‐sperm bundles were collected from multiple colonies (listed below) and separated using 60 μm mesh filters. Sperm was set aside, and eggs were washed again 3× in 60 μm mesh in 0.2 μm FSW. Two types of reproductive crosses were generated. Individual crosses were produced from colonies that were spawning on the same night to maximize the chance of gamete compatibility following a diallel design using roughly equal numbers of eggs and a sperm concentration of 10^6^/ml. A bulk culture was also produced by mixing eggs and sperm from multiple colonies together with eggs from parents: Ytag1, A, Z1, D, C, D4, Ytag2, C3, A1, D5, D2, C1, and sperm from parents: Ytag1 Z3, C2, WT3, C3, Y2, H, C1, A, C, D4). Gametes were monitored for cell division and when successful cell division was observed, embryos were washed 3× in 0.2 μm FSW to remove residual sperm and maintained at a density of ~1 larvae/mL in individual culture tanks with constant flow through of 27.5°C FSW. The bulk culture was reared in a single 500 L tank with 27.5°C FSW. Simultaneously, three crosses from four parental colonies (hereafter larval families) were reared in individual 45 L tanks with flow through 27.5°C FSW. Following spawning, tissue biopsies from contributing parents were preserved in 100% EtOH and stored at −20°C for DNA extraction and ITS2 amplicon sequencing.

### 2.3 Larval Thermal Stress Experiment

At 4 days post‐fertilization, individual larvae from each cross (HxWT3, WT3xC1, H1xWT3) and the bulk culture were placed into individual wells of sterile Corning 3.5 mL 48‐well plates pre‐filled with ~3 mL of the same SeaSim FSW in the holding tanks (Corning, Sigma‐Aldrich). Plates were sealed into individual transparent plastic bags to prevent evaporation and placed into temperature‐ and light‐controlled incubators set at 27°C and 32°C. The same bags were used throughout the experiment and only opened during larval measurement period. Well plates were not cleaned due to no algal overgrowth, and no larval changes were required as there was no evaporation due to the bags sealing the well plates. Light was set at 60 PAR (μmol m^−2^ s^−1^, Sylvania FHO24W/T5/865 fluorescent tubes) with a 14:10 light: dark cycle. For the bulk culture, four technical replicate 48‐well plates were placed at each temperature, each with 48 larvae (*n* = 192 larvae at 27°C and 32°C each). For the individual crosses, three technical replicate plates with two rows per cross in each plate were placed at each temperature (*n* = 16 larvae of each cross/plate × 3 crosses × 3 plates = 144 larvae at 27°C and 32°C in total). At the time of well‐plate stocking, a subset of different individual larvae from both the bulk culture and individual crosses were preserved in 100% EtOH and stored at −20°C for DNA extraction and ITS2 amplicon sequencing. The experiment continued for 2 months with periodic screening of larvae for survival and collection of non‐invasive physiological data. The experiment was terminated when survival at 32°C reached an average of 50% across all treatments to ensure sufficient remaining biomass for analysis of symbiont community composition. Surviving larvae were individually preserved in 100% EtOH and stored at −20°C for DNA extraction and amplicon sequencing.

### 2.4 Juvenile Thermal Stress Experiment

At 4 days post‐fertilization, additional larvae from both the bulk culture and individual crosses were placed into sterile 6‐well plates at 10 larvae per well with anywhere from 1 to 5 larvae settling into juveniles in (Corning, Sigma‐Aldrich) pre‐filled with SeaSim FSW. A small chip (1 mm^2^) of coral rubble was added to each well as a settlement inducer. Larvae were allowed to settle overnight followed by a complete water change. Due to high variability in settlement success, only bulk culture larvae settled into juveniles in high enough numbers for adequate replication in the subsequent thermal stress experiments. As with the larval experiment, the 6‐well plates were individually wrapped in plastic bags and placed into temperature and light incubators with SeaSim FSW that was not changed throughout the experiment. The experiment continued for 20 days with periodic monitoring of juveniles for survival and collection of non‐invasive physiological data. The experiment was then terminated when survival at 32°C reached an average of 50%, and samples were preserved as described for larvae. Finally, since multiple juveniles may have settled from larvae in one well, we performed a general linear mixed effects model to test the effects of well and plate localization on mortality of juveniles using a Poisson distribution with mortality being predicted by the variables “well” and “plates.”

### 2.5 Physiological Data

Larval survival was scored from 19 April 2018 to 15 June 2018 over 21 timepoints from 0 to 57 days after the experimental start. Juvenile survival was scored from 31 May 2018 to 20 June 2018 over 3 timepoints. Larvae were deemed dead when they were not seen inside the well. A juvenile was deemed dead when tissue was not present and only skeleton remained.

Larval size was measured on 23 May 2018, between 30 and 39 days from the experiment start (timepoints 17 and 18). To measure size, wells were visualized under a dissecting microscope (Zeiss, Switzerland) and a still image was captured when larvae were in a lateral position. Finally, the scale of the images was captured using a calibration rule to the photos and the software ImageJ was used to measure the size.

Microscopy Pulse Amplitude Modulated fluorometry (Microscopy PAM, Walz, Germany) was used to assess the photosynthetic performance and functioning of symbionts within individual *M. digitata* larvae and juveniles. Specifically, effective photosystem II quantum yield (YII = Fm′ − F/Fm′) was measured at two timepoints: 17 and 53 days. ImagingWin software (v2.46x6) was used with the following settings: Measuring light (Intensity 6, Frequency 8), Actinic light (Intensity 3), Gain and Damping (both 2). Individual larvae were outlined as the area of interest (AOI). Two replicate larvae per plate for 3× replicate plates were measured (*n* = 6 larvae per cross per treatment per timepoint).

### 2.6 Statistical Analysis of Physiological Data

Physiological data was modeled as a function of temperature treatment within crosses using R (v. 4.1). For all models, assumptions of homogeneity of variance, linearity, normality and autocorrelation were checked, and tests modified where appropriate. The presence (1) or absence (0) of larvae and juveniles at each monitoring time‐point was used as input for a survival analysis using the “Survminer” package. Survival over time was modeled as a function of temperature treatment and cross type. Significance of factors was assessed using a Generalized Linear model using a binary (logit) distribution. Larval size was modeled as a function of length and temperature treatment with a random effect of their well plate environment using the model (Length ~ Treatment, random = ~1|Plate, data = larvae size, na.action = na.exclude) in the “lme” package. For the PAM fluorometry data, linear mixed models were fit for each timepoint using the package “nlme” to test for significant differences in YII due to temperature treatment (categorical factor) and replicate plate (random factor). The main effect of temperature was determined using Type II Analysis of Deviance tests using the “car” package. The assumption of homogeneity of variance was violated for PAM values originating from the bulk culture larvae at 57 days; therefore, a generalized least squares model (GLS) was implemented using the “nlme” package, including an ARMA correlation structure (“plate”) with categorical weights (VarInd) for temperature treatment, fit by maximizing the restricted log‐likelihood (REML).

### 2.7 DNA Extraction, Preparation of ITS2 Amplicon Libraries, and Sequencing

DNA was extracted from adult biopsies, skeletal debris, and individual larvae and juveniles using modified SDS protocol outlined here (Quigley, Willis, and Bay 2017). The Symbiodiniaceae ITS2 region was amplified using the SYM_VAR_5.8S2 (forward: 5′‐GTGACCTATGAACTCAGGAGTCGAATTGCAGAACTCCGTGAACC‐3′) and SYM_VAR_REV (reverse: 5′‐CTGAGACTTGCACATCGCAGCCGGGTTCWCTTGTYTGACTTCATGC‐3′) primers following the protocol described in Hume et al. (2019). Most PCR reactions contained: 0.5 μL of equimolar (10 mM) dNTPs, 5 μL 5X Q5 PCR buffer (New England Bio Labs), 0.25 μL Q5 DNA polymerase (New England Bio Labs), 2 μL DNA (20 ng/μl) template, 0.25 μL of each primer (10 μM), 0.25 μL BSA (20 mg/μl) (New England Bio Labs), and 16.5 μL Milli‐Q ultrapure water per reaction. However, some samples with limited DNA were amplified at 1ul DNA with an adjusted amount of 17.5 μl Milli‐Q ultrapure water per reaction). The amplification profile included an initial denaturation step of 98°C for 30 s with cycles of 98°C for 10 s, 56°C for 1 min, and 72°C for 30 s, with a final elongation step of 72°C for 5 min. Samples were amplified to the lowest cycle number at which a band was first observed (Table S1). Twelve samples failed to amplify and were discarded from subsequent steps. Water was used as a negative control during PCR and only samples from reactions where no negative control bands were observed were carried forward for sequencing. In a second six cycle PCR, Illumina primers with custom dual index barcodes were incorporated. Barcoding PCR reactions contained: 0.5 μL of equimolar (10 mM) dNTPs, 5 μL 5X Q5 PCR buffer (New England Bio Labs), 0.25 μL Q5 DNA polymerase (New England Bio Labs), 3 μL DNA template, 1 μL of each of two barcodes (10 μM), 0.25 μL BSA (20 mg/μl) (New England Bio Labs), and 14 μL Milli‐Q Ultrapure water per reaction. The amplification profile for barcoding contained an initial denaturation step of 98°C for 30 s and six cycles of 98°C for 10 s, 59°C for 1 min, and 72°C for 30 s, with a final elongation step of 72°C for 2 min. Barcoded amplifications were cleaned using a SPRI bead based size selection protocol (Beckman Coulter) with a 0.65:1 bead to sample ratio to select for nucleic acid sequences between 300 and 400 bp in length, which corresponds with target Symbiodiniaceae ITS2 band sizes (Hume et al. 2019). Following bead clean up and size selection samples were pooled in groups of 4–10 based on visual assessment of gel band intensity, and a 1:100 dilution of each sample pool was qPCR amplified (Aria MX, Agilent) using the Illumina i5 and i7 sequencing primers to quantify the relative abundance of each sample for subsequent pooling. qPCR reactions were run in duplicate. Pools were then mixed in equimolar volumes, and the final library was sequenced at the USC NCCC Molecular Genomics Core on the Miseq V2 sequencing platform (PE 250). Samples with insufficient read depth (*N* = 36, < 1000 reads per sample) were subject to two rounds of additional amplification, barcoding, size selection, and sequencing to increase read yields (Table S1). Additional samples with sufficient read depth in the first sequencing run were also re‐amplified to serve as internal controls for composition and relative abundance of ITS2 amplicons across independent runs. High similarity was evident within internal control samples across sequencing runs (Figure S1), justifying concatenation of raw reads from individual runs by sample prior to statistical analyses.

### 2.8 Assigning Taxonomy

A hybrid Symportal‐DADA2 approach was used to delineate and taxonomically identify sequence variants. An important attribute of Symportal is that it measures symbiont abundance based on the formation of ITS2 profiles that correspond to “defining intragenomic variants” (DIVs) from the family Symbiodiniaceae (Hume et al. 2019). This assignment accounts for potential overinflation of diversity metrics by adjusting for intragenomic variation of ITS2 Amplicon Sequence Variant (ASVs) using patterns of co‐occurrence across samples (Hume et al. 2019). However, given the high relatedness of our sample set, co‐occurrence more likely results from the biological mechanism of shared inheritance. The Symportal pipeline also discards background symbiont types, defined as those sequences that are represented by ≤ 200 reads. However, Symportal does not account for repeated or paired measures, and is therefore susceptible to missing background variants present initially in low abundance but increasing in abundance in time‐series sample sets. Therefore, to understand whether rare variants with increased abundance in heat‐treated samples were also present in control and pre‐stress conditions regardless of their absolute threshold abundances in addition to correcting for the presence of intragenomic variation when assessing community diversity, Symportal DIVs and taxonomies were overlaid onto DADA2 ASVs. To do this, a Symportal analysis was used to generate a taxonomy database specific to this dataset and to identify similar co‐occurring sequences that comprise representative DIVs. DADA2 was then run on the same raw read dataset to generate ASVs that were then assigned sequence and DIV identities based on the Symportal taxonomy. This approach also allowed us to retain background‐low abundance reads while also accounting for intragenomic variation of ITS2 using the Symportal framework as described in greater detail below.

Amplicon sequencing of the ITS2 region was undertaken across 120 samples with 90 samples being successfully resulting in 4.76 × 10^6^ raw reads. These reads were processed by Symportal with default parameters (“‐‐analyze” with “‐‐num_proc 3”). This yielded a DIV matrix and taxonomy assignments for each sample based on the Symportal database. The taxonomy database from this run was used to generate a Phyloseq data bin for downstream analysis.

In parallel, the same reads were trimmed of their Illumina adaptors using bbduk (Bushnell 2020), and their barcodes using cutadapt (Martin 2011; Table S2). These reads were then filtered and processed using DADA2, resulting in a total of 4.08 × 10^6^ trimmed reads (Callahan et al. 2016). A total of 81 of 90 samples passed the trimming and filtering steps in DADA2. The heat‐treated larval family samples were lost in this processing due to poor‐quality reads (Table S2). An ASV table was generated using the standard DADA2 protocol for ITS amplicon sequencing reads (Callahan et al. 2016). Taxonomy was assigned to ASVs by using the Symportal database specific to this dataset as described above to ensure a 1:1 correspondence. ASVs were then collapsed into DIVs by summing counts across individual ASVs comprising the DIV using the multisequence ITS2‐profile types identified by Symportal. Rare ASVs (those originally discarded by Symportal) were retained as individual columns in the matrix. For example, in our dataset there were four *Cladocopium* ASVs that formed four unique and overlapping ITS2 DIV profiles, “C15,” “C15‐C15dq,” “C15‐C15dq‐C15dr” and “C15‐C15dq‐C15dr‐C_1365.” For samples that Symportal identified as having “C15” or “C15‐C15dq” as their dominant DIV, the DADA2 ASV matrix was modified to create a new column which was the sum of all C15 ASVs or all C15 plus C15dq ASVs, effectively replacing the individual ASV columns with the DIV. The majority of samples had “C15‐C15dq‐C15dr‐C_1365” as their dominant DIV. Counts for samples that exhibited a C15 or C15 + C15dq DIV but still had individual counts for C15dq or C15dr were also maintained in the sequence matrix (Table S3).

### 2.9 Alpha and Beta Diversity

The Shannon Diversity Index was calculated in Phyloseq (McMurdie and Holmes 2013) as a metric of alpha diversity for each sample. Shannon diversity was then modeled as a function of temperature treatment (bulk culture larvae and juveniles) or larval family using beta regression (Cribari‐Neto and Zeileis 2010) to be able to properly fit continuous values between 0 and 1. For families, pre‐stress and ambient larvae were grouped together. Pairwise comparisons among means with respect to factor levels (pre‐stress, ambient, and heat, or larval family) were conducted using Tukey's HSD, adjusting *p*‐values for multiple comparisons. Beta diversity was visualized using nonmetric multidimensional scaling (NMDS) with a Bray‐Curtis distance implemented in Phyloseq for bulk culture larvae and juveniles and larval families (McMurdie and Holmes 2013). Following NMDS scaling of symbiont communities, evenness of dispersion across bulk culture larvae and juveniles and larval families was evaluated using the betadisper function in the vegan package (Oksanen et al. 2007). Only bulk juvenile samples satisfied assumptions for parametric testing (Table S4), and a PERMANOVA was used to test the effect of treatment on community differences using the adonis2 function. A non‐parametric test implemented with the anosim function was used to test the effect of treatment (bulk culture larvae) or family on beta community differences (Oksanen et al. 2007).

## 3 Results

### 3.1 Fitness‐Related Traits Vary With Temperature in Larvae and Juveniles

**FIGURE 1:**
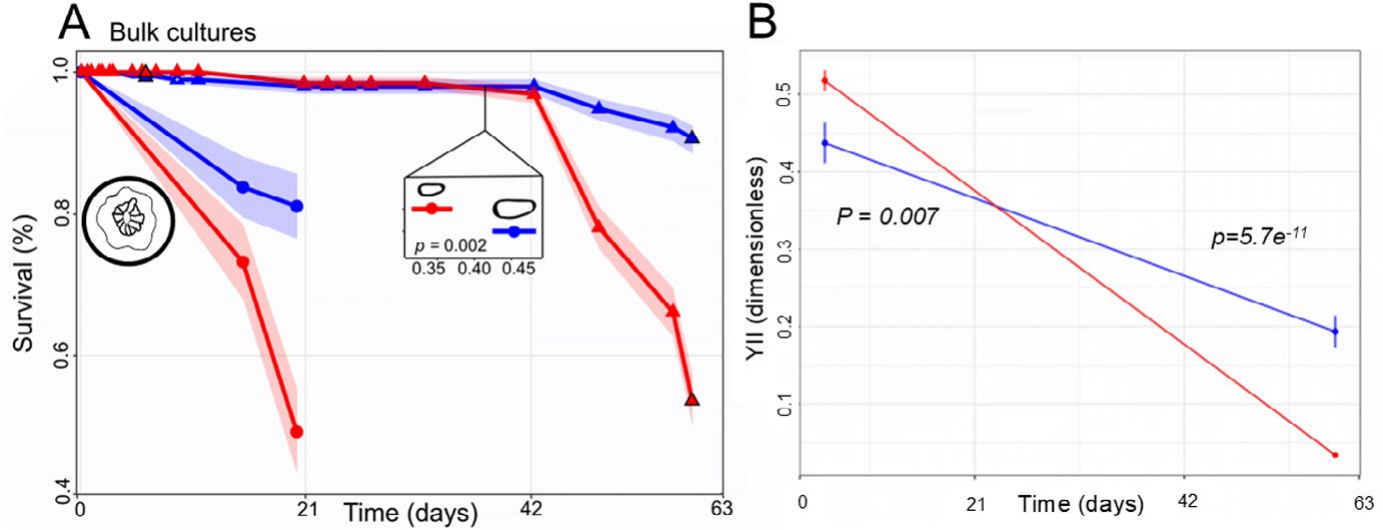
Survival, size and photosynthetic efficiency of ambient and heat‐treated bulk culture larvae and juveniles. (A) Lines indicate the percent survival of juveniles (circles) and larvae (triangles) as a function of treatment (red: Heat 32°C, blue: Ambient 27°C). Shading indicates the 95% confidence interval. Differences in the size of ambient and heat exposed bulk culture larvae were quantified at one time point and are shown as the mean ± standard error as a function of treatment in the inset. (B) Larval photosynthetic efficiency (YII) was measured at two timepoints (17 and 53 days).

There were no significant effects of well or plate on juvenile mortality indicating that increased juvenile mortality was not attributable to having multiple individuals in some wells (Table S5). Larvae and juveniles produced from bulk culture fertilizations survived significantly better at 27°C compared to those exposed to 32°C (Figure 1, Kaplan–Meier survival, *p <* 0.0001 and 0.00014, respectively). Compared to larvae, juveniles exhibited a higher susceptibility to heat, with a mean survival of ~20 days, whereas larvae did not reach a mean of 50% survival until ~57 days of exposure to 32°C (Figure 1a and Figure S2).

Larvae produced from bulk culture fertilizations were significantly smaller at 32°C (0.45 ± 0.03 mm) compared to those at 27°C (0.34 ± 0.03 mm) after 34 days of heating (LRT, df = 1, *p =* 0.002, Figure 1A). After 17 days in treatment temperatures, bulk larvae at 32°C had significantly higher photosynthetic yields relative to larvae at 27°C (Figure 1B, *p =* 0.007). This was reversed after 53 days, in which larvae at 27°C had significantly higher yields (Figure 1B, *p =* 5.7e^−11^).

### 3.2 Fitness‐Related Traits Vary Among Larval Families

**FIGURE 2:**
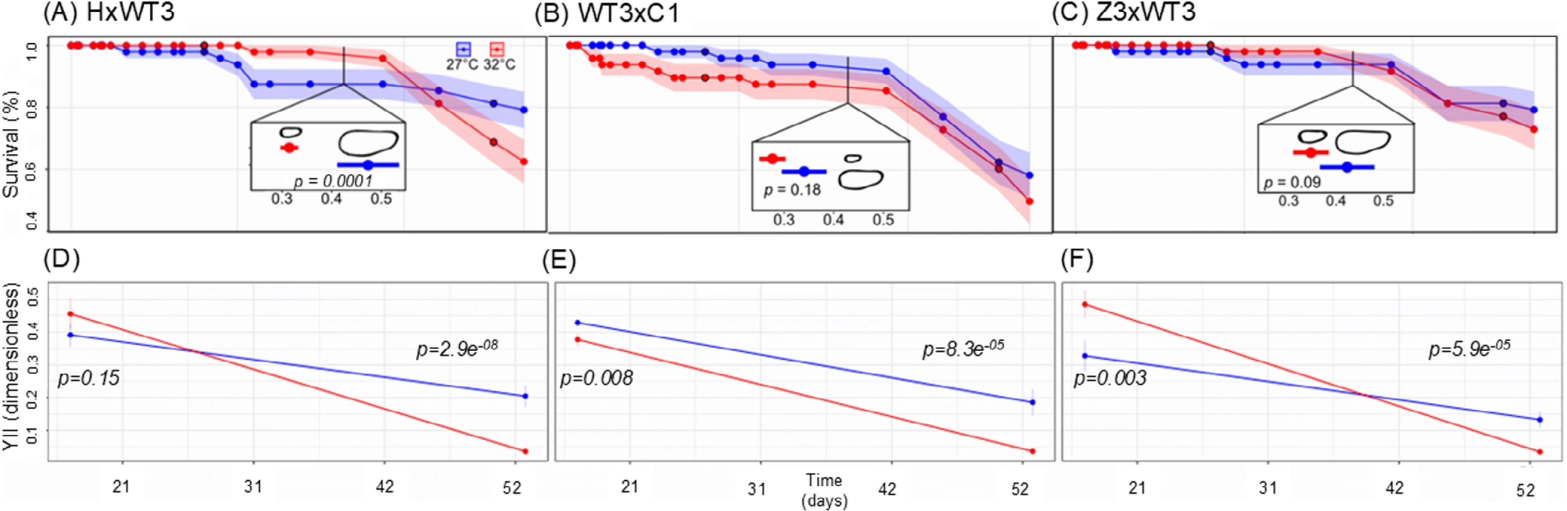
Survival, size and photosynthetic efficiency of ambient and heat‐treated larvae derived from crosses between adults (A) HxWT3, (B) WT3xC1, and (C) Z3xWT3. Within panels A–C lines indicate the percent survival of larvae as a function of treatment throughout the duration of the experiment (red: Heat 32°C, blue: Ambient 27°C). Shading indicates the 95% confidence interval. Differences in the size of ambient and heat‐treated larvae were quantified at one time point and are shown as the mean ± standard error as a function of treatment in the central insets in panels A–C. Photosynthetic efficiency (YII) of ambient and heat‐treated larvae derived from crosses between adults (D) HxWT3, (E) WT3xC1, and (F) Z3xWT3 was measured at two timepoints (17 and 53 days).

**FIGURE 3:**
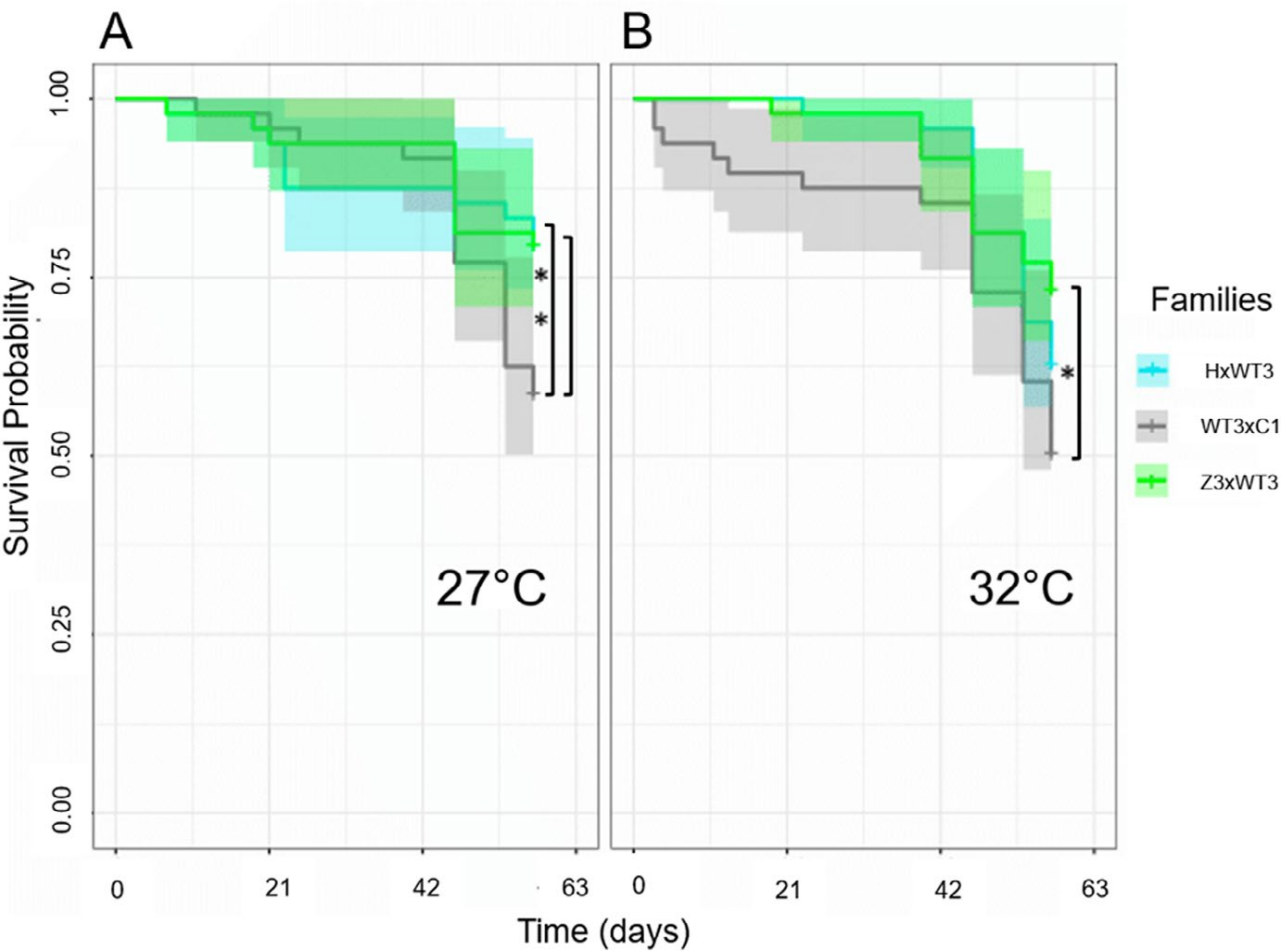
Survival probabilities of ambient and heat‐treated larvae from crosses between adults HxWT3 (light blue), WT3xC1 (gray), and Z3xWT3 (light green). (A) Survival probabilities for family comparisons HxWT3 and WT3xC1 and Z3xWT3 and WT3xC1 were significantly different in ambient conditions (27°C) at 53 days (*p* = 0.045, *p* = 0.042, respectively). (B) Survival probabilities for family comparisons Z3xWT3 and WT3xC1 were significantly different in heat‐treated conditions (32°C) at 53 days (*p* = 0.025).

Larval survival was impacted by family origin (HxWT3, WT3xC1, and Z3xWT3, Figure 2). Specifically, larvae from family Z3xWT3 survived significantly better at both temperatures compared to larvae from family WT3xC1 (Kaplan–Meier survival—KM, *p =* 0.042 and 0.025). Larvae from HxWT3 survived significantly better compared to WT3xC1 larvae at 27°C but not 32°C (KM, *p =* 0.045 and 0.18), but survival was similar compared to Z3xWT3 at both temperatures (KM, *p =* 0.97 and 0.35). Hence, at 32°C, survival of larvae was highest overall in the family Z3xWT3, followed by HxWT3 and WT3xC1 (Figure 3). Larval survival from the three crosses was not significantly different at 27°C compared to 32°C after 57 days of heating (Kaplan–Meier survival, *p =* 0.12, 0.4, and 0.5, for HxWT3, WT3xC1, and ZxWT3, respectively, Figures 2 and 3).

On average, effective quantum yield of photosystem II (YII) in larval families ranged from 0.49 ± 0.1 to 0.03 ± 0.009. YII varied significantly at both 17 and 53 days between temperature treatments for all families except HxWT3. Specifically, YII values measured in larvae from families HxWT3, WT3xC1, and Z3xWT3 were all significantly greater at 27°C (0.2 ± 0.08–0.13 ± 0.06) compared to 32°C (0.04 ± 0.01–0.03 ± 0.008) after 53 days of heating (Likelihood Ratio Test‐LRT, df = 1, *p =* 2.9e^−08^, 8.3e^−05^, 5.9e^−05^, respectively, Figure 2A–C). In family WT3xC1, YII values were significantly greater in larvae at 27°C compared to 32°C (0.4 ± 0.03–0.38 ± 0.05, LRT, df = 1, *p =* 0.008) following 17 days of exposure, although the opposite trend was found in Z3xWT3 (0.3 ± 0.1–0.5 ± 0.1, LRT, df = 1, *p =* 0.003). Similar to the bulk culture (Figure 1), larvae from family Z3xWT3 exhibited significantly higher YII at 32°C (0.52 ± 0.01) compared to those at 27°C (0.44 ± 0.03) after 17 days of heating (LRT, df = 1, *p =* 0.007). After 57 days, this trend was significantly reversed (0.19 ± 0.02 versus 0.03 ± 0.002, Generalized Least Squares—GLS, df = 1, *p =* 5.7e^−11^).

Larval size also varied across the three families at 27°C, in which HxWT3 larvae were the largest (0.49 ± 0.06 mm, Figure 2A). After 34 days of heating at 32°C, larvae from all families were smaller, although only larvae from family HxWT3 were significantly smaller than their paired controls (0.32 ± 0.02 mm length; *n* = 6, LRT, df = 1, *p =* 0.003, Figure 2A). Larvae from the family WT3xC1 were the smallest after heating (0.28 ± 0.03 mm compared to 0.34 ± 0.05, LRT, df = 1, *p =* 0.18). The decrease in larval size under heat was less in family Z3xWT3 (32°C: 0.35 ± 0.04 mm, 27°C: 0.42 ± 0.06, LRT, df = 1, *p =* 0.09).

### 3.3 Symbiont Communities Differ in Larvae, But Not Juveniles Exposed to Heat Stress

**FIGURE 4:**
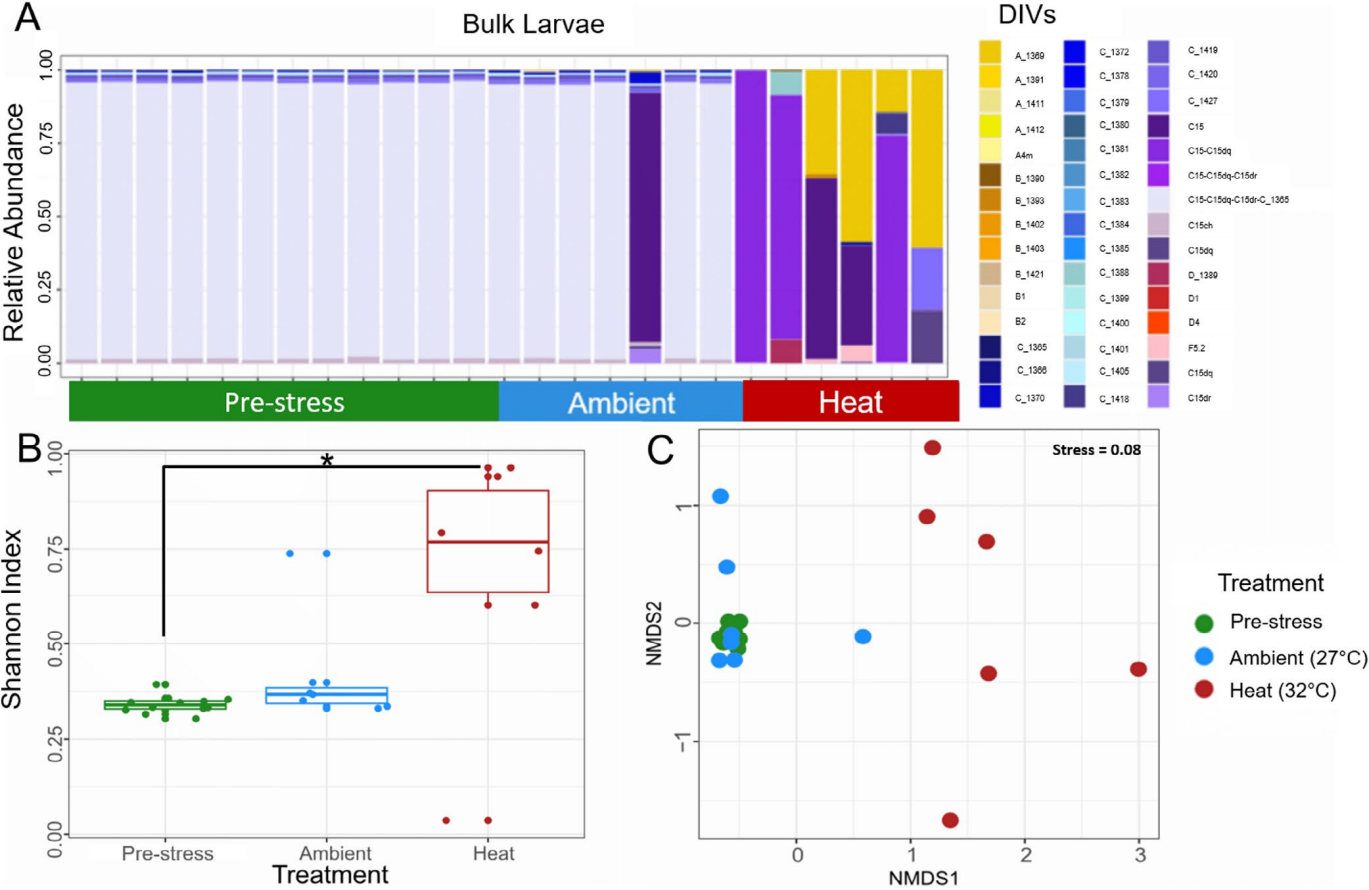
Symbiont community composition in bulk larval cultures. (A) Barplots showing relative abundance of Symbiodiniaceae DIVs from pre‐stress (green), ambient (27°C, blue) and heat (32°C, red) treated bulk larvae. (B) Boxplot distributions of Shannon Alpha diversity in pre‐stress, ambient (27°C) and heat (32°C) treated larvae. The asterisks indicate significant pairwise differences between pre‐stress (green) and heat (32°C, red) treatment (*p < 0.05). (C) Beta diversity of symbiont communities in pre‐stress, ambient and heat‐treated bulk larvae visualized using an NMDS plot with Bray‐Curtis distance (Stress = 0.08).

Larvae produced from bulk culture fertilizations did not exhibit differences in their Symbiodiniaceae communities between 27°C and the pre‐experimental timepoint (or pre‐stress). Control and pre‐stress larvae were dominated by the ITS2 DIV profile C15‐C15dq‐C15dr‐C_1365 and low abundances (< 5%) of *Symbiodinium* spp. were also detected (Figure 4A and Figures S4 and S5). Larvae from the 32°C treatment showed a markedly different symbiont community compared to larvae at 27°C. This difference included both a change in the identity to one of the four dominant C15 ITS2 DIV profile identified for this dataset (Figure S4) as well as notable increases in relative abundance of *Symbiodinium* spp., *Breviolum* spp., *Dursudinium* spp., and *Fugacium* spp. in addition to other *Cladocopium* spp. variants (Figure 4A).

Shannon diversity, which in this case describes the number and relative abundance of algal DIVs within an individual larva (Peet 1974), also confirmed the shift in community composition within heat‐treated larvae compared to pre‐stress samples (pairwise comparisons between pre‐stress and 32°C, *p =* 0.0033, Figure 4B and Table S6). A similar trend is evident between heat and ambient treatments (pairwise comparisons between 27°C and 32°C, *p =* 0.0535, Figure 4B and Table S2). Beta diversity, which describes differences in symbiont community composition among larval samples, also distinguished heat‐treated larvae from ambient and pre‐stress samples (NMDS with Bray‐Curtis distance, Figure 4C and Table S7). Additional testing of dispersion between beta diversity community values revealed a significant effect of treatment on larval community composition (BETADISPER *p <* 0.001, ANOSIM, *R =* 0.48 and *Sig =* 0.001, Table S8).

**FIGURE 5:**
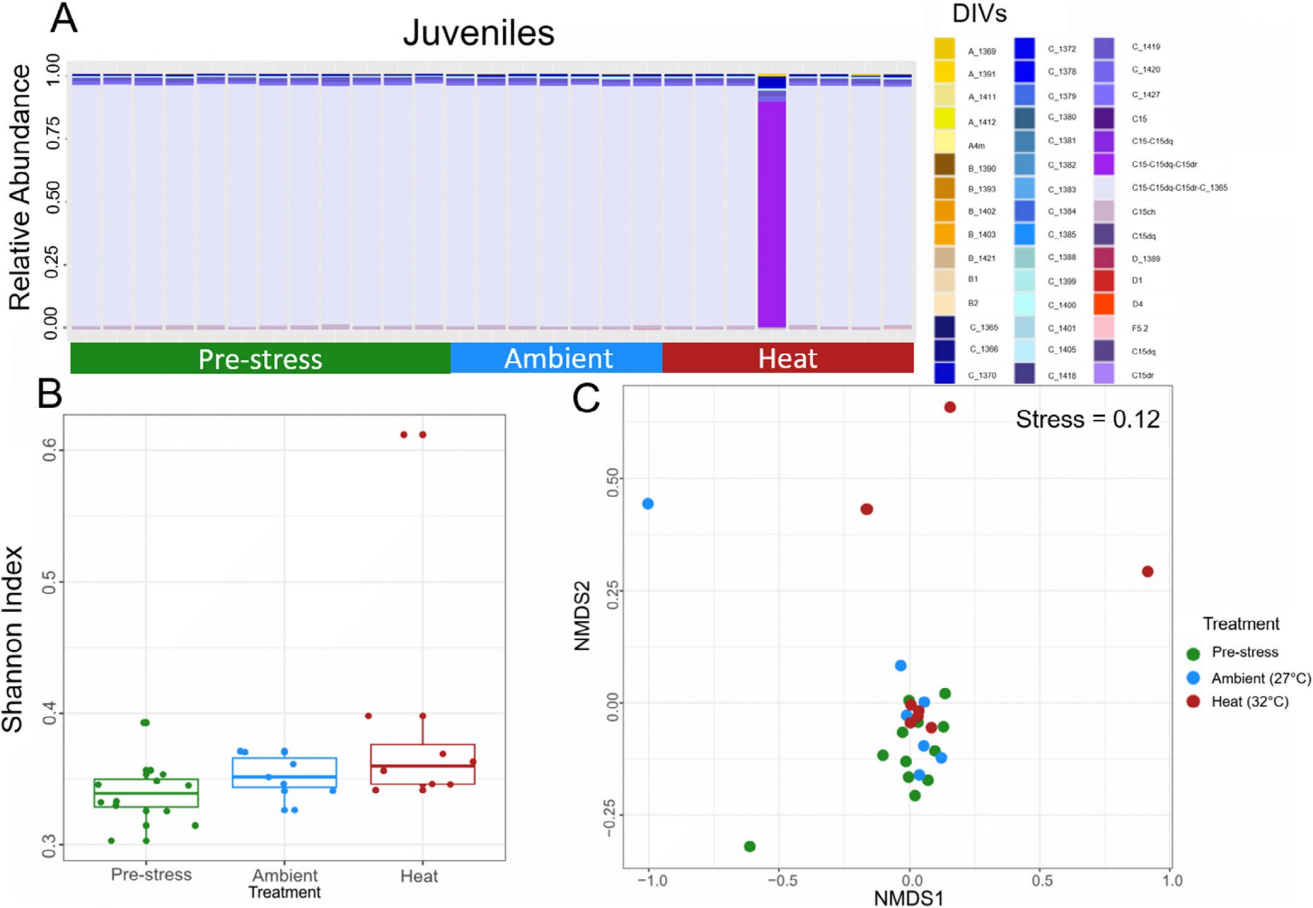
Symbiont community composition in juveniles produced from bulk cultures. (A) Barplot showing relative abundance of Symbiodiniaceae DIVs in pre‐stress (green), ambient (27°C, blue) and heat (32°C, red) treated juveniles. (B) Boxplot distributions of Shannon Alpha diversity in pre‐stress, ambient (27°C) and heat (32°C) treated juveniles. (C) Beta diversity of pre‐stress larvae, ambient and heat‐treated juveniles visualized using an NMDS plot with Bray‐Curtis distance (Stress = 0.12).

This pattern was not evident in juveniles derived from the same bulk culture fertilization and exposed to a similar heat stress. Juveniles remained dominated by the same C15‐C15dq‐C15dr‐C_1365 DIV regardless of treatment (Figure 5). Although some heat‐treated juveniles showed increases in *Symbiodinium* spp. and *Breviolum* spp. variants in comparison to ambient controls and pre‐stress larvae, no difference in alpha diversity was detected between ambient and heat treatments (pairwise comparisons between 27°C and 32°C *p =* 0.29, Figure 5A,B and Table S6). A similar trend was detected between pre‐stress larvae and heat‐treated juveniles as was observed between ambient and heat‐treated larvae (pairwise comparisons between pre‐stress larvae and 32°C, *p =* 0.059, Figure 5B and Table S6). Visualizing beta diversity using NMDS with Bray‐Curtis distance confirmed no major differences in symbiont communities between the pre‐stress, ambient, and heat‐treated juvenile samples (Figure 5C). Nor was a significant effect of treatment detected on beta community dispersion (BETADISPER *p =* 0.946, PERMANOVA *p =* 0.385, Table S9).

### 3.4 Targeted Reproductive Crosses Reveal Distinct Symbiont Communities in Mothers Are Reflected in Offspring

**FIGURE 6:**
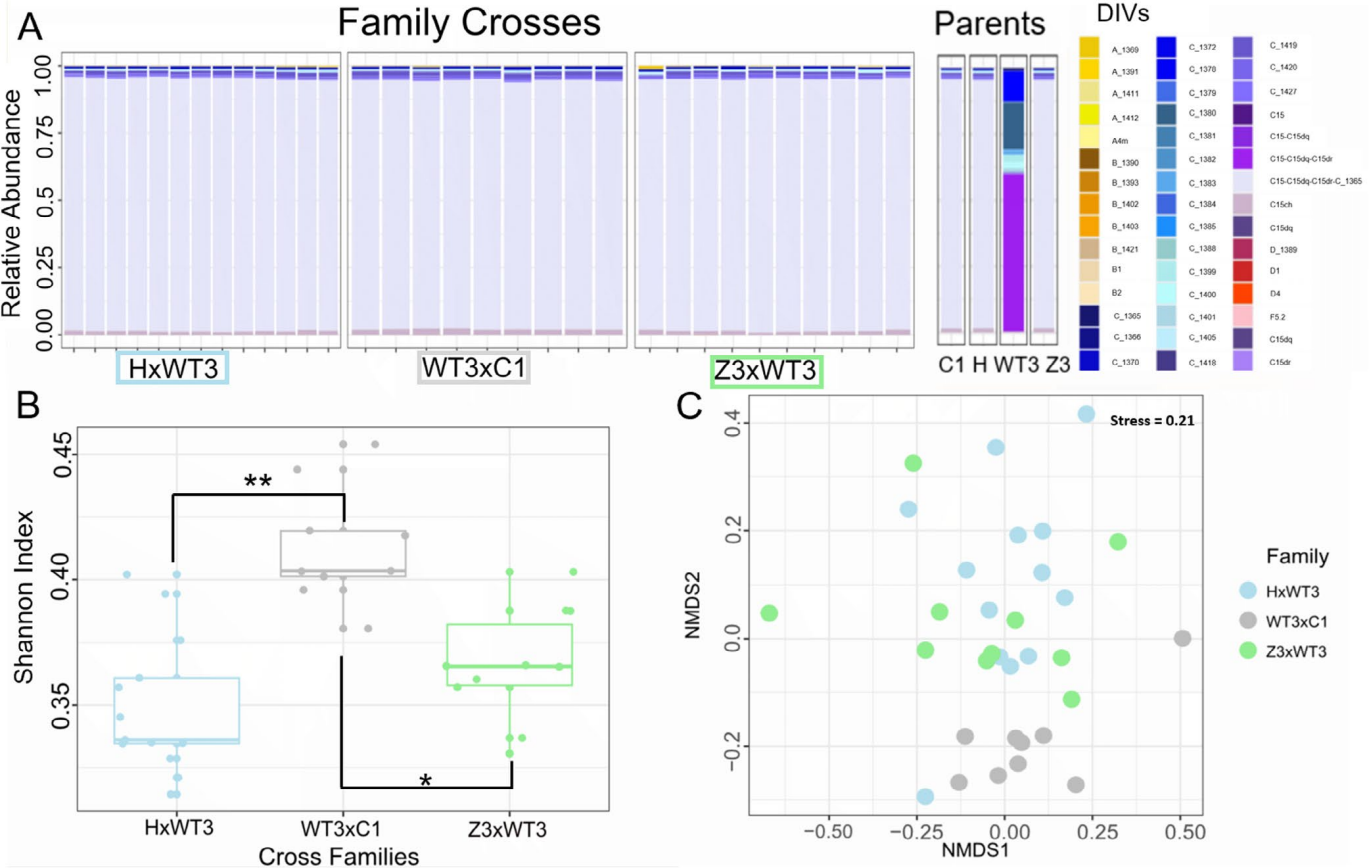
Symbiont community composition in larval crosses and their parents in control conditions. (A) Barplot showing relative abundance of Symbiodiniaceae DIVs in individual larvae grouped by family (notation: Dam × sire). Symbiodiniaceae DIVs of parent coral are shown in an additional panel. (B) Boxplot distributions of Shannon Alpha diversity. Asterisks indicate significant pairwise differences between family pairs, Z3xWT3 and WT3xC1 (*padj = 0.0001), and HxWT3 and WT3xC1 (**padj < 0.0001). (C) Beta diversity of symbiont communities by family visualized using an NMDS plot with Bray‐Curtis distance (Stress = 0.21).

Only larvae from the 27°C treatment yielded successful amplification, restricting comparisons to differences among families in the ambient treatment. Larvae from all three crosses were dominated by the same *Cladocopium* spp. DIV (C15‐C15dq‐C15dr‐C_1365). Background taxa, or those present at < 5% abundance, exhibited a DIV profile that was similar to the bulk culture larvae at 27°C (Figures 4A and 6A). Parental colonies were similarly dominated by DIV C15‐C15dq‐C15dr‐C_1365, except for colony “WT3,” which was dominated by *Cladocopium* profile C15‐C15dq‐C15dr (Figure 6A).

Alpha diversity was significantly higher in family WT3xC1 in comparison to the other crosses (HxWT3—WT3xC1, *padj* < 0.0001, and WT3xC1—Z3xWT3, *padj* = 0.0001, Figure 6B). This pattern was also reflected in beta diversity (NMDS with Bray‐Curtis distance, BETADISPER *p* = 0.017, Anosim, *R =* 0.17 and *Sig =* 0.006; Table S10). Specifically, larvae from cross WT3xC1 clustered strongly together, likely driven by low‐abundance DIV profiles, whereas the other crosses were more evenly distributed across the ordination space (Figure 6C and Table S6).

## 4 Discussion

In this study, we demonstrate changes in symbiont communities in the early life‐history stages of a common, vertically transmitting coral, *Montipora digitata*, in response to heat stress. Specifically, we found symbiont communities differed between temperature treatments in larvae but detected no differences in juveniles derived from the same bulk culture fertilization. Although we cannot confirm whether these changes in larvae are due to active (i.e., shuffling by the host animal) or passive (i.e., increase in opportunistic symbionts or differential susceptibility among symbiont community members) mechanisms, we did observe significant differences in survival duration between larvae and juveniles, with larvae surviving more than twice as long as juveniles. Moreover, symbiont communities in heat‐stressed larvae became dominated by representatives of canonically stress‐tolerant genera. Finally, we also show that increased maternal symbiont diversity is reflected in offspring. Overall, our results indicate that larvae can survive twice as long when compared to juveniles under the same warming conditions, potentially driven by symbiont shuffling. This suggests that the juvenile life stage may be more at risk from climate warming due to limited flexibility.

### 4.1 Life Stage Specific Differences in Physiology and Symbiont Community Diversity in Response to Thermal Stress

Although both larvae and juveniles were dominated by the same C15 DIV, larvae survived much longer on average and their symbiont community composition showed greater diversity in the heat treatment compared to juveniles. There is ample evidence to show that symbiont communities drive host physiology in coral adults and to a lesser extent offspring (Quigley et al. 2022, 2023; Terrell et al. 2023), underpinned by differences in symbiont tolerance to stress (Swain et al. 2021). *Symbiodinium microadriaticum* and *Durusdinium trenchii*, for example, tend to produce less reactive oxygen species (ROS) in culture, a molecular response associated with coral bleaching, when exposed to heat stress, compared to *Breviolum minitum* and *Cladocopium goreaui* (Lesser 2019). Additionally, these dynamics within hosts can start as early as gametogenesis in vertically transmitting species, as changes in symbiont communities within oocytes of the same species were detected after a mass bleaching year (Quigley, Willis, and Kenkel 2019). Taken together, we postulate that the altered symbiont community in larvae of the vertically transmitting *M. digitata* may have afforded them a fitness advantage which then allowed them to persist longer under heat stress.

Assuming the symbiont community shift led to direct gains in heat tolerance in larvae, this suggests that either the maternal colonies or the larvae have the capacity to actively rearrange their symbiont communities (as an acclimatization mechanism) to cope with heat stress. In horizontally transmitting coral species, the capacity to shuffle symbiont communities in response to heat stress events has been shown in juvenile (Terrell et al. 2023) and adult life stages (Berkelmans and Van Oppen 2006; Ross et al. 2013), indicating an acclimatory response to heat stress is available to some corals. Similar studies are limited for vertically transmitting coral species. Work on another congener, *Montipora capitata*, provides insight into this capacity. *M. capitata* is a vertically transmitting coral in which individuals generally host either *Cladocopium* (which are more susceptible to thermal stress), *Durusdinium* (which are less susceptible to thermal stress), or some combination of both (Cunning, Silverstein, and Baker 2018; Dilworth et al. 2021). However, no changes to symbiont communities were observed for either *M. capitata* colonies when exposed to short‐term stress (Dilworth et al. 2021). More recently, however, corals of this species previously and recently sampled along heat stress extremes sites along K āne‘ohe Bay showed mixed communities of *Durusdinium* and *Cladocopium* that changed in dominance and with stress levels (de Souza et al. 2022), perhaps indicating the capacity to shuffle in response to a sufficiently intense environmental effect. A short‐term heat stress did not result in symbiont shuffling in early life stages of *M. captitata*; however, there is a capacity to inherit symbiont communities that resemble the dominant community present in the parental coral (Harris et al. 2022), a unique feature of vertically transmitting coral (Harris et al. 2022; Quigley, Willis, and Kenkel 2019). Taken together, these results suggest that extreme heat and vertical transmission (Bright and Bulgheresi 2010) can lead to heritable changes in symbiont community composition in vertical transmitting corals over generations.

Indeed, this was the case in *M. digitata* , where symbiont communities in oocytes were observed to change in parallel to their maternal sources in response to a mass bleaching event (Quigley, Willis, and Kenkel 2019). Here we expand on these findings to show that *M. digitata* larvae are also able to change their symbiont communities in response to a thermal stress. Alternatively, juveniles lacked this capacity, which is more aligned with the fixed symbiont communities observed in later developmental stages of other vertically transmitting corals. More work is needed to disentangle symbiont establishment and winnowing compared to the mechanisms of shuffling and switching. Finally, although these changes in the symbiont communities appear to be acclimatory, we cannot conclusively determine if these changes preceded differential mortality and so cannot tease apart the influence of these two processes.

Alternatively, larvae may be more robust compared to juveniles for reasons unrelated to symbionts. Larvae may be more resistant generally because of their positive buoyancy from high lipid content early in life, which exposes them to harsh environmental conditions such as high ultraviolet radiation and temperature at the sea surface (Glynn 1993; Wellington and Fitt 2003; Rodriguez‐Lanetty, Harii, and Hoegh‐Guldberg 2009; Aranda et al. 2011; Gleason and Hofmann 2011). Moreover, during the motile larval stage, they are actively exposed to both surface and benthic conditions from several days to multiple weeks (Ritson‐Williams et al. 2009), forcing them to withstand highly variable environmental conditions. Metamorphosis is also an energetically costly process that depletes larval energy reserves and may result in more susceptible juvenile stages (Edmunds, Gates, and Gleason 2001; Ritson‐Williams et al. 2009). The enhanced survival of the larvae compared to juveniles under heat stress may therefore result from either or both the change in symbiont community and an overall robustness of larvae. In summary, we hypothesize that lower survival of juveniles may be driven by lack of an ability to adjust symbiont communities combined with diminished energetic reserves post‐metamorphosis, suggesting the juvenile stage may be the most susceptible life‐history stage for corals.

Interestingly, we found Symbiodiniaceae community composition in *M. digitata* juveniles to be highly stable regardless of heat exposure. This is in contrast to other studies in which shuffling in juvenile corals has been repeatedly confirmed during initial symbiont acquisition (Little, van Oppen, and Willis 2004; Yorifuji et al. 2017; Cumbo, Baird, and van Oppen 2013), and through development (Quigley et al. 2017, 2020; Terrell et al. 2023), only stabilizing later in life (Abrego et al. 2008). These changes through juvenile ontogeny are generally referred to as winnowing (Abrego et al. 2008). Symbiont communities can change during this winnowing period and are characterized by increases in the abundance of a diversity of symbionts. As part of this, some opportunistic symbionts can be taken up; but communities generally stabilize through time to resemble adult communities, either due to competition or initiation of immune responses (McIlroy et al. 2019; Abrego et al. 2008). Although we saw community differences in *M. digitata* larvae when exposed to heat stress, the contrasting stability of symbiont communities in juveniles under the same conditions suggests that winnowing in this species occurs in larvae and is fixed after this life stage. This further reinforces the notion that the juvenile stage may be the most susceptible to stress. Further work is needed to characterize the dynamics of the symbiosis during this important ontogenetic transition.

### 4.2 Variation in Fitness Among Larval Families and the Role of Symbiont Community Diversity

To better understand parental contributions to fitness differences, we undertook controlled genetic crosses. Previous work in this species showed a concordance between symbiont communities in parents and their eggs (Quigley, Willis, and Kenkel 2019), and we were expecting similar patterns in larvae—as we indeed observed here. Symbiont community is a heritable trait (Quigley, Willis, and Bay 2017; Quigley, Willis, and Kenkel 2019), which implies that at least some familial effects will be present. This has been demonstrated in a number of species in the Indo‐Pacific ( *Acropora tenuis* and *Montipora digitata* , Quigley, Willis, and Bay 2017; *Seriatopora hystrix* , Quigley et al. 2018). Importantly, we also showed that differences in symbiont communities among families are associated with differences in survival in larvae. In particular, larvae from cross WT3xC1 exhibited lower average survival and had the most disparate background symbiont community. This may have been due to the maternal influence of WT3, which had a distinct endosymbiont community dominated by C15‐C15dq‐C15dr when compared to the other three parental colonies. Therefore, poorer offspring survival could be due to increased abundance of opportunistic *Cladocopium* spp. variants (Howe‐Kerr et al. 2020). We lacked the ability to measure the degree to which symbiont community differences among families can change in response to heat stress due to low sample sizes. It may be that an increase in potentially opportunistic symbionts would increase or decrease the capacity for coral early life stages of *M. digitata* to alter their performance under heat stress.

## 5 Conclusion

Overall, we observed changes in symbiont communities in the early life stages of *Montipora digitata* in response to heat stress. We could not determine if these changes were due to an active or passive mechanism. However, our results suggest juvenile stages of *M. digitata* are more susceptible to heat stress compared to the larval stage. To determine if shuffling is indeed an active acclimatory mechanism, higher‐resolution time series sampling of early life stages should be conducted. As rare taxa increased in abundance in larvae that were heat stressed, future studies should also examine the degree to which background symbiont communities can be inherited, which will require larger cross designs. As *M. digitata* is a vertically transmitting species, the degree to which rare‐heat tolerant species are inherited in offspring may be an indicator of their future resistance to heat stress, which will play a critical role in their survival in a rapidly changing climate.

## Author Contributions

**Daniel Olivares‐Cordero:** conceptualization (equal), data curation (equal), formal analysis (equal), investigation (equal), methodology (equal), project administration (equal), visualization (equal), writing – original draft (equal), writing – review and editing (equal). **Carly D. Kenkel:** conceptualization (equal), data curation (equal), formal analysis (equal), funding acquisition (equal), investigation (equal), methodology (equal), project administration (equal), resources (equal), supervision (equal), writing – review and editing (equal). **Courtney Timmons:** data curation (equal), methodology (equal), resources (equal). **Kate M. Quigley:** conceptualization (equal), data curation (equal), formal analysis (equal), funding acquisition (equal), investigation (equal), methodology (equal), project administration (equal), resources (equal), supervision (equal), visualization (equal), writing – original draft (equal), writing – review and editing (equal).

## Conflicts of Interest

The authors declare no conflicts of interest.

## Supporting information


Data S1.

Table S1.

Table S2.

Table S3.

## Data Availability

A preprint has been uploaded to EcoEvoRxiv under doi: https://doi.org/10.32942/X2H91R. All raw sequence reads can be found in SRA project PRJNA1112848 with a token link and will be released in June of 2025. Metadata and code can be found in our Github, Danny1196/Monti_Shuff(github.com) and through the Open Science Framework, https://osf.io/n5dsx/?view_only=438f16a143b6433aa5fd69a3ac067f0d.
