# Supplementary material for: Symbiont Community Changes Confer Fitness Benefits for Larvae in a Vertically Transmitting Coral
Source: Ecol Evol. 2025 Jan 12;15(1):e70839. doi: 10.1002/ece3.70839 (PMC11725385; doi:10.1002/ece3.70839)
Supplement: Supplementary file 1 — Data S1. [file ECE3-15-e70839-s002.docx]

**Supplementary Tables and Figures**

Life-stage specific differences in survival are associated with greater shifts in symbiont community composition in larvae of the vertically transmitting coral, *Montipora digitata*

Daniel Olivares-Cordero^,^* , Courtney Timmons^1^, Carly D. Kenkel^1^, Kate M. Quigley^2, 3^

^1^Department of Biological Sciences, University of Southern California, Los Angeles CA, USA

^2^ Minderoo Foundation, Perth, WA 6009, Australia

^3^James Cook University, Townsville, Australia


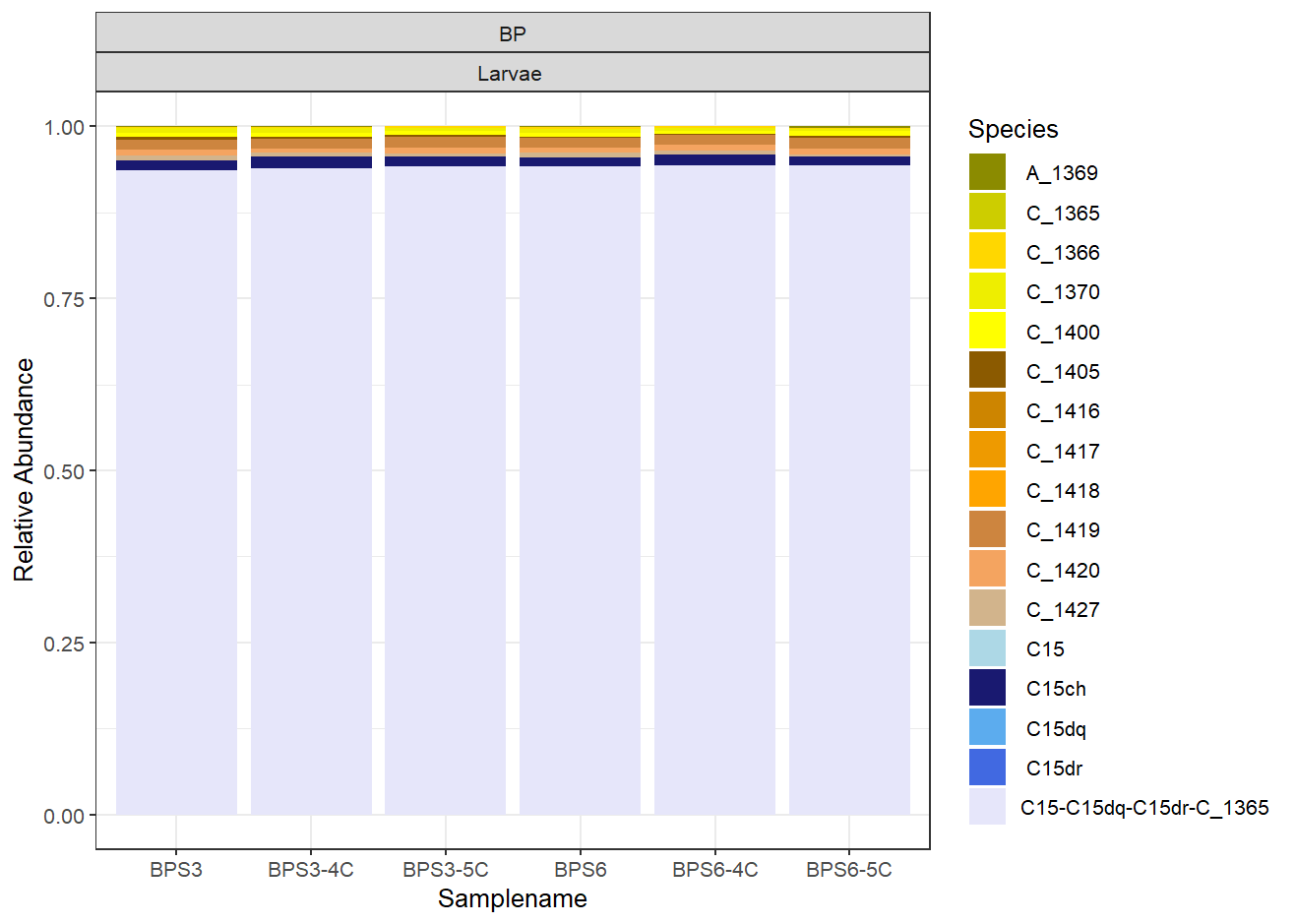


**Supplementary Figure S1.** Relative abundance plot for internal controls from three different sequencing runs. Samples with numbers represent the month of their sequencing run (4=April, 5=May). The “C” indicates that they are a replicate control. Samples without the “C” designation, indicate the data is from the original sequencing run.


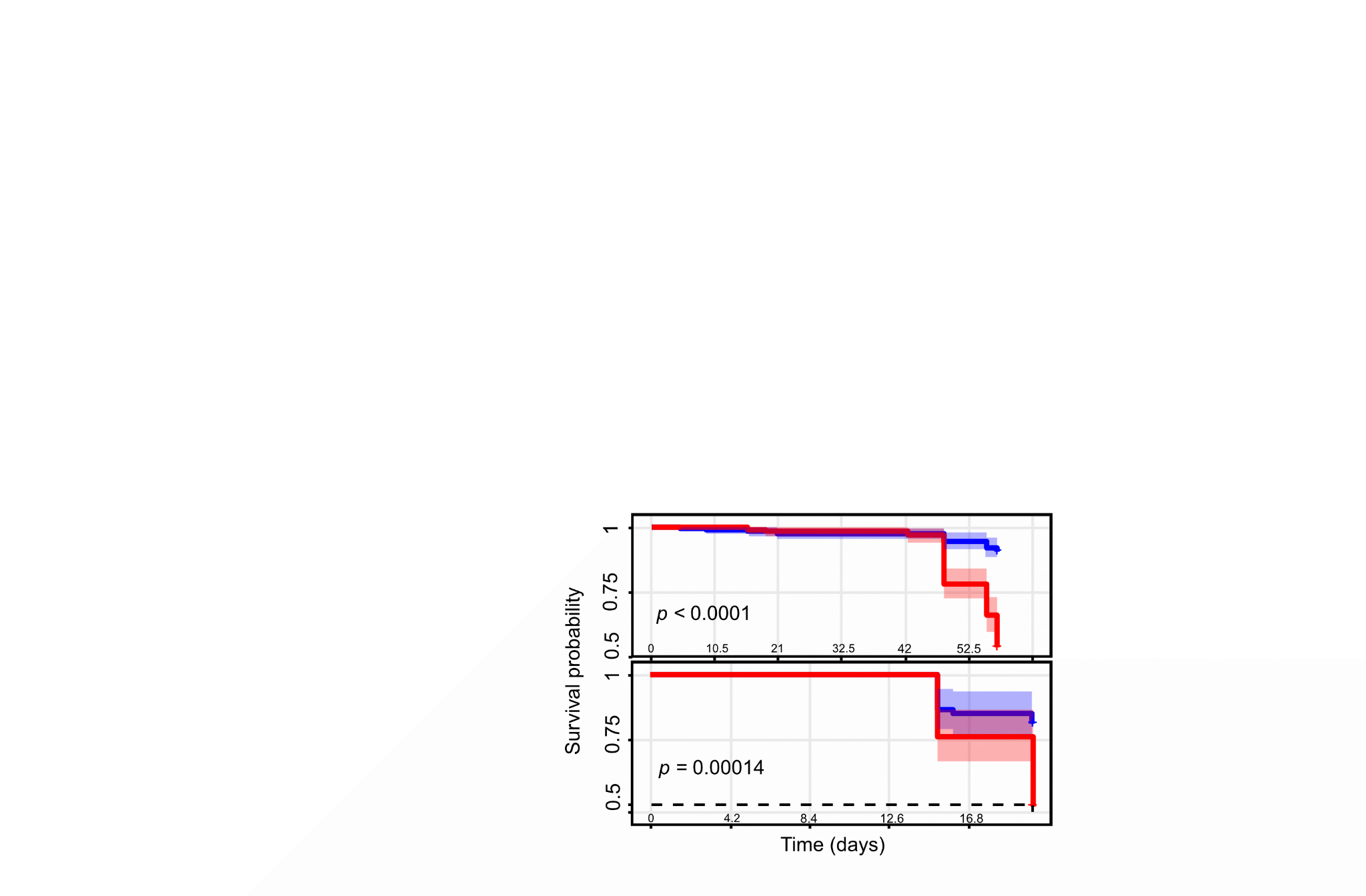


**Supplementary Figure S2.** Survival probabilities for bulk larvae (top) and bulk juveniles (bottom) in ambient and heat stressed treatments.


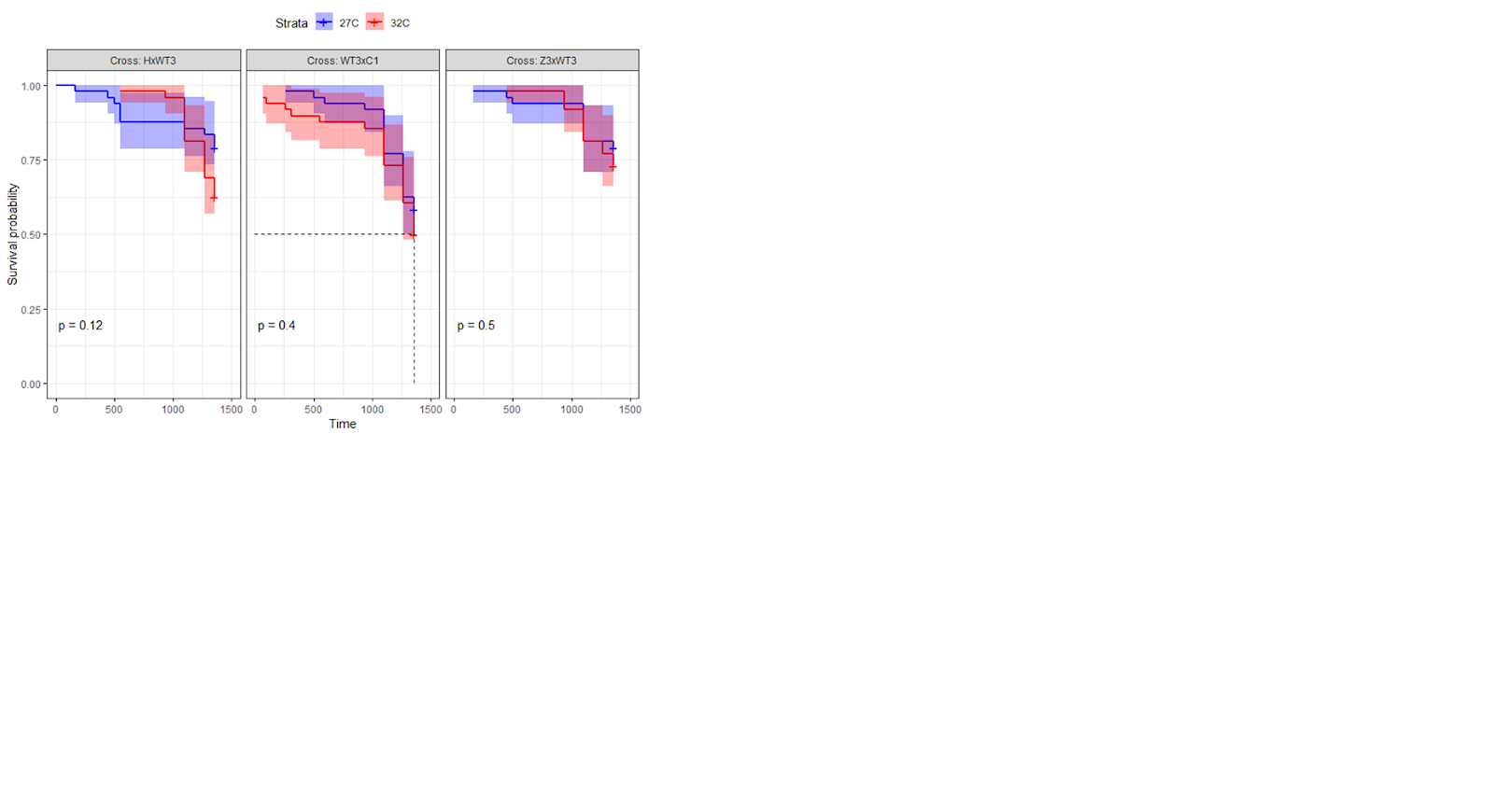


**Supplementary Figure S3.** Survival probabilities for cross larvae in ambient and heat stressed treatments.


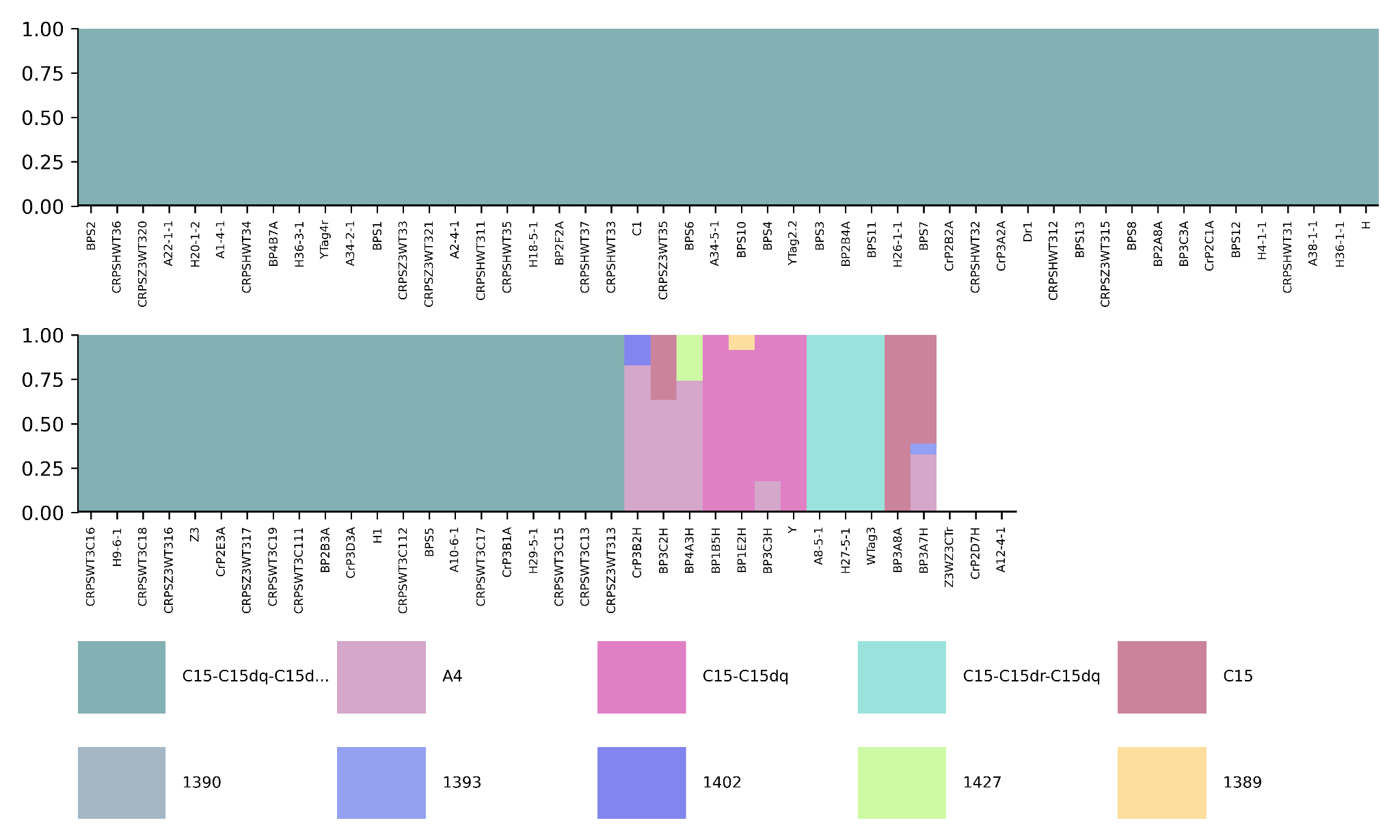


**Supplementary Figure S4.** Symportal-generated DIV Profile abundance plot by sample.


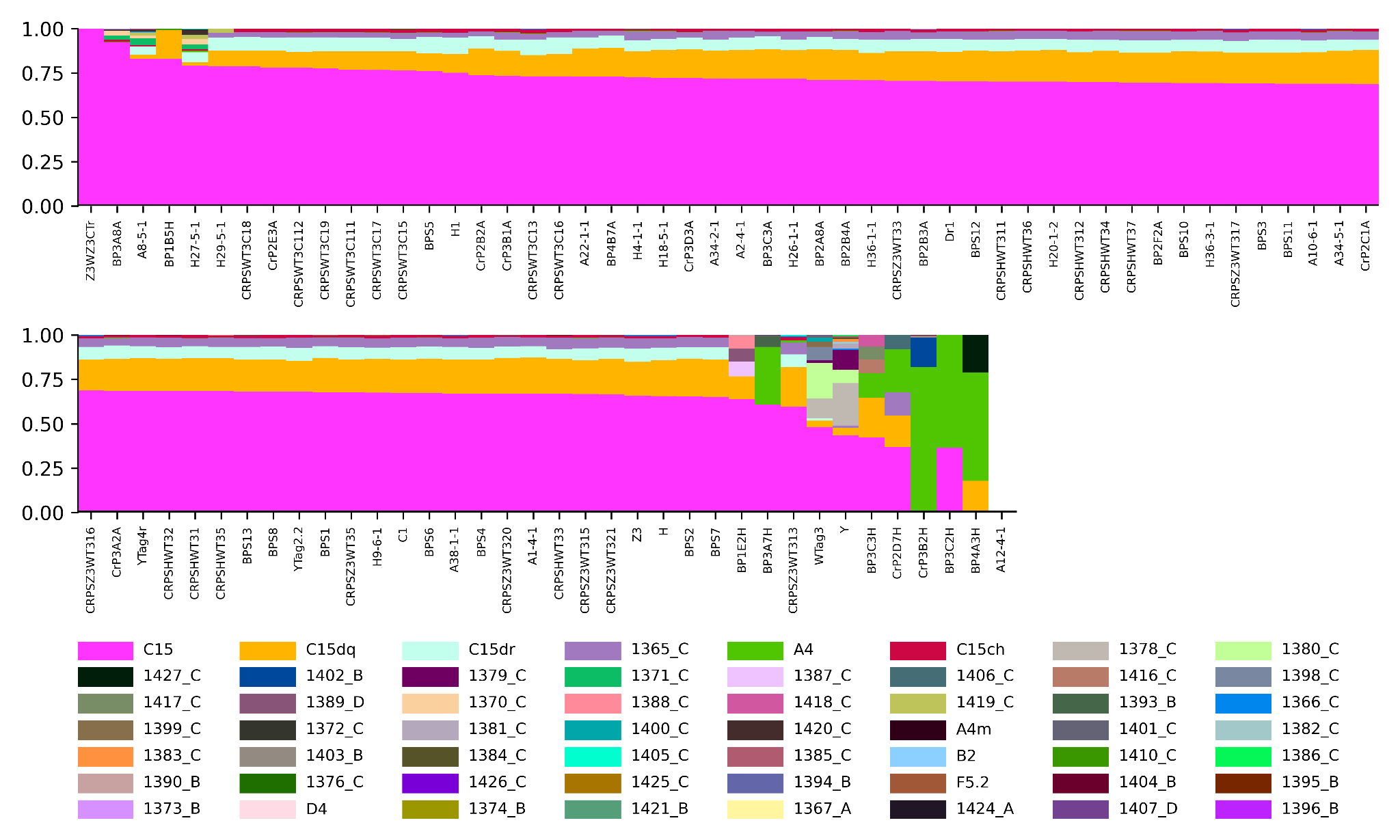


**Supplementary Figure S5.** Symportal generated amplicon variant abundance plot by sample.

**Supplementary Table S4.** Permutation Test for homogeneity of dispersions (Betadisper) for Bulk Larvae, Bulk Juveniles and Cross Family Larvae.

|  |  | df | Sum of Sqs | Mean Sq | F | Permutations | Pr(>F) |
| --- | --- | --- | --- | --- | --- | --- | --- |
| Bulk Larvae | Groups | 2 | 0.097 | 0.049 | 11.69 | 999 | 0.001*** |
| Bulk Larvae | Residual | 22 | 0.091 | 0.004 |  |  |  |
| Bulk Juveniles | Groups | 2 | 0.0007 | 0.0004 | 0.059 | 999 | 0.946 |
| Bulk Juveniles | Residual | 24 | 0.159 | 0.006 |  |  |  |
| Cross Family Larvae | Groups | 2 | 0.025 | 0.012 | 4.41 | 999 | 0.017* |
| Cross Family Larvae | Residual | 30 | 0.084 | 0.003 |  |  |  |

**Supplementary Table S5.** Results from a generalized linear mixed effects model estimating the random effects well and plate localization had on juvenile mortality.

Formula: glm(J$Alive ~ J$well + J$Plate, family = poisson, data =J)

| Coefficient | Estimate | Std. Error | Z value | Pr(>\|z\|) |
| --- | --- | --- | --- | --- |
| Intercept | -0.2343 | 0.170 | -1.376 | 0.169 |
| J$well | 0.0037 | 0.032 | 0.117 | 0.907 |
| J$Plate | 0.0006 | 0.005 | 0.140 | 0.889 |

**Supplementary Table S6.** Pairwise testing of Shannon diversity means in bulk larvae, bulk juveniles and family larvae.

| Groups | Contrast | Estimate | SE | df | z.ratio | p.value |
| --- | --- | --- | --- | --- | --- | --- |
| Bulk Larvae | Ambient-Heat | -0.25 | 0.1 | Inf | -2.32 | 0.0535 |
| Bulk Larvae | Ambient-Prestress | 0.06 | 0.09 | Inf | 0.64 | 0.7990 |
| Bulk Larvae | Heat-Prestress | 0.31 | 0.09 | Inf | 3.25 | 0.0033 |
| Bulk Juveniles | Ambient-Heat | -0.037 | 0.025 | Inf | -1.5 | 0.29 |
| Bulk Juveniles | Ambient-Prestress | 0.012 | 0.022 | Inf | 0.56 | 0.84 |
| Bulk Juveniles | Heat-Prestress | 0.049 | 0.022 | Inf | 2.28 | 0.059 |
| Family Larvae | HxWT3-WT3xC1 | -0.06 | 0.009 | Inf | -6.39 | <0.0001 |
| Family Larvae | HxWT3-Z3xWT3 | -0.02 | 0.009 | Inf | -2.09 | 0.09 |
| Family Larvae | WT3xC1-Z3xWT3 | 0.04 | 0.01 | Inf | 4.17 | 0.0001 |

**Supplementary Table S7.** NMDS Stress values tables for goodness of model

| Group Tested | All Samples | Bulk Larvae | Bulk Juveniles | Family Crosses |
| --- | --- | --- | --- | --- |
| Stress Value | 0.088 | 0.08 | 0.12 | 0.21 |
| Dimensions | 2 | 2 | 2 | 2 |

**Supplementary Table S8.** Analysis of Similarities for Bulk Larvae based on treatment

|  | R | Significance | Permutations |
| --- | --- | --- | --- |
| Bulk Larvae based on Treatment | 0.48 | 0.001 | 999 |

**Supplementary Table S9.** Permanova for Bulk Juveniles based on treatment.

|  | df | Sum of Sqs | R2 | F | Pr(>F) |
| --- | --- | --- | --- | --- | --- |
| Treatment | 2 | 0.04 | 0.08 | 1.07 | 0.385 |
| Residual | 24 | 0.43 | 0.92 |  |  |
| Total | 26 | 0.47 | 1.0 |  |  |

**Supplementary Table S10.** Analysis of Similarities for Cross Larvae based on treatment

|  | R | Significance | Permutations |
| --- | --- | --- | --- |
| Bulk Larvae based on Treatment | 0.17 | 0.006 | 999 |
